# Rasch analysis of the self-reported PedsQL™ 4.0 Generic Core Scales by Australian children

**DOI:** 10.1186/s12955-025-02441-4

**Published:** 2025-12-15

**Authors:** Joseph Kwon, Rakhee Raghunandan, Son Hong Nghiem, Kirsten Howard, Emily Lancsar, Elisabeth Huynh, Martin Howell, Stavros Petrou, Sarah Smith

**Affiliations:** 1 Nuffield Department of Primary Care Health Sciences, University of Oxford, Oxford, England; 2 The Leeder Centre for Health Policy, Economics and Data, School of Public Health, University of Sydney, Sydney, Australia; 3 Centre for Health Services Research, University of Queensland, Brisbane, Australia; 4 Department of Health Services Research and Policy, Australian National University, Canberra, Australia; 5 Department of Health Services Research and Policy, London School of Hygiene and Tropical Medicine, London, England

**Keywords:** Psychometrics, Rasch measurement, Health-Related quality of life, Pediatric quality of life inventory

## Abstract

**Purpose:**

The Pediatric Quality of Life Inventory™ Version 4.0 Generic Core Scales (PedsQL GCS), comprising 23 items covering four subscales (physical, emotional, social, and school functioning), is a widely applied generic measure of childhood health-related quality of life (HRQoL). This study aimed to assess the psychometric performance of the self-reported Child version in an Australian general population of children using psychometric criteria based on Rasch measurement theory.

**Methods:**

To minimise type I error, a random sample of *n* = 500 was drawn from 1,874 Australian children aged 11–12 years who participated in the Longitudinal Study of Australian Children and completed the PedsQL GCS Child self-report measure. The partial credit model was applied. The following properties were assessed: unidimensionality, model goodness of fit, model reliability, targeting, item fit, threshold ordering, differential item functioning (DIF), and local dependency.

**Results:**

Areas of poor performance included: weak support for unidimensionality of school functioning subscale; only emotional functioning subscale showing sufficient model goodness of fit; poor targeting, suggesting that item thresholds are limited in discriminating HRQoL differences in this cohort; seven items showing disordered thresholds; and five pairs of items being locally dependent. Only two items showed poor fit, and there was no evidence of non-uniform DIF regarding sex and age.

**Conclusion:**

Not all Rasch measurement criteria were satisfied by the PedsQL GCS Child self-report measure applied in Australian children. This Rasch-based psychometric evaluation identified potential areas of improvement that can complement the known conceptual, practical, and classical psychometric strengths of the PedsQL GCS.

**Supplementary Information:**

The online version contains supplementary material available at 10.1186/s12955-025-02441-4.

## Background

The use of patient-reported outcome measures (PROMs) to assess health-related quality of life (HRQoL) in childhood populations (aged ≤ 18 years) can facilitate clinical research and practice, but also presents methodological challenges compared to their application in adult populations [1–3]. One key challenge is to account for age-based biopsychosocial development during childhood, which alters the health dimensions of relevance across childhood age groups [4–6]. The feasibility of child self-report and appropriate design features (e.g., number of response levels and pictorial features) is also affected by age at completion [1].

The Pediatric Quality of Life Inventory 4.0 Generic Core Scale (PedsQL GCS) is a generic HRQoL questionnaire representing four core subscales of health and role functioning as delineated by the World Health Organisation: physical, emotional, social, and school [7]. It is one of the most widely used measures of childhood HRQoL, with over 90 translations and multiple disease-specific supplementary modules [3, 8, 9]. There are different versions of the PedsQL GCS defined by target age groups: Toddler for age 2–4 years; Young Child for 5–7 years; Child for 8–12 years; and Teen for 13–18 years. All versions have the same four subscales and 23 items, and are compatible with child self-report and proxy-report, except for the Toddler module, which has two fewer school items and is compatible only with proxy-report [3, 7]. Coverage of all childhood age groups except infancy (< 2 years of age), continuity across age groups in the core conceptual constructs measured, applicability to both clinical and general childhood populations, high practicality in taking less than four minutes to complete on average, and availability of over 90 translations are key strengths of the PedsQL GCS [9].

Psychometrics is concerned with the extent to which a set of items (questions) operates as a ruler (measure) with sufficient measurement properties [10–12]. Key ‘classical’ psychometric properties include content validity, reliability, construct validity, responsiveness, and patient and investigator burden [1, 10–15]. Selection of a PROM for clinical research and practice should involve evaluating its performance against established criteria for these properties in the intended target [8, 16–18]. To that end, the PedsQL GCS has frequently been assessed for its psychometric performance according to classical criteria in diverse general and clinical childhood populations and has shown acceptable performance [19–24]. Overall, the PedsQL GCS possesses key strengths in terms of conceptual coverage, practicality, and classical psychometric performance.

Psychometric analyses based on Rasch measurement theory can supplement the classical approach [25]. The Rasch paradigm provides a powerful set of diagnostic statistics by which to evaluate the performance of the measure [25, 26]. In the Rasch model, the probability of a respondent endorsing a particular item response is assumed to be a logistic function of the relative distance between his or her position on the latent scale and the position of the item response on the scale [25]. Conducting psychometric analyses within the Rasch paradigm overcomes several limitations of the classical approach. Notably, it creates scores that are truly interval (as opposed to ordinal) to enable parametric analyses [27, 28]. It also allows for invariant comparison of mean PROM scores (i.e., scores are independent of the sample and scale from which they are derived) within and across populations [28]. Rasch-based methods generate error terms that are individual rather than group-based, therefore enabling the scores to be used for individual-level comparisons (such as to monitor an individual patient’s progress) rather than only to compare the average of two groups. They also provide a powerful means of identifying areas for potential improvement, including parts of the scale where content may be missing, items that do not work well, response options that are not working as intended, and items that work differently (i.e., are biased) in relation to different respondent groups [29]. Rasch-based psychometric evaluations have thus been applied to existing and newly developed HRQoL measures and multi-attribute utility instruments [30–35].

Previous evaluations have generally found mixed performance for the PedsQL GCS based on Rasch psychometric criteria, satisfying some while failing to meet others. Specifically, PedsQL GCS has been evaluated on: Korean schoolchildren aged 8–18 years [36]; Singaporean preschool children with refractive errors aged 3–6 years [37]; children with life-threatening conditions in the US aged 2–18 years [38]; Iranian schoolchildren aged 8–18 years [39]; Canadian paediatric cancer patients aged 2–17 years [40]; male children with Duchenne muscular dystrophy (DMD) in the US aged 5–16 years [41]; and a multinational cohort of children with DMD aged 3–18 years [42]. No previous work has used Rasch-based methods to evaluate the PedsQL GCS in Australian children. Given the emphasis by the paediatric PROM research community on obtaining self-reported data from children wherever feasible [1, 15, 43], this study aims to evaluate performance of the PedsQL GCS on Rasch-based measurement criteria according to self-reported data. The work was a secondary analysis as part of a larger project that used a representative sample of a general population of Australian children aged 11–12 years.

## Methods

### Data: Child health checkpoint

The Child Health CheckPoint study (henceforth, ‘CheckPoint’) contains the relevant data for this study, details of which can be found elsewhere [44, 45]. Briefly, the CheckPoint study consisted of physical health and biomarker collection from one of two nationally representative cohorts of Australian children (Cohort B) participating in the Longitudinal Study of Australian Children (LSAC) [46–49]. This cohort had been recruited into the LSAC study aged 0–1 years (initial *N* = 5,000) in 2003-04, followed by a data collection wave every two years. The CheckPoint cross-sectional survey was conducted between Waves 6 and 7 in 2015 when the cohort B children were aged 11–12 years. This contained self-reported data from 1,874 children for the Child version of the PedsQL GCS.

### Pediatric quality of life inventory 4.0 Generic Core Scale

The PedsQL GCS Child version has 23 items across four subscales: physical functioning with eight items; emotional functioning with five items; social functioning with five items; and school functioning with five items [7]. The item wording is detailed in Table 1. All items share the same question root and a five-level ordinal response scale (0=‘Never’, 1=‘Almost never’, 2=‘Sometimes’, 3=‘Often’, and 4=‘Almost always’).

**Table 1:** Item wording for pediatric quality of life inventory 4.0 generic core scale child self-report version

**Question root**: “In the past ONE month, how much of a PROBLEM has this been for you…”				
**Response levels**: 0 = Never; 1 = Almost never; 2 = Sometimes; 3 = Often; 4 = Almost always^a^				
Item #	Physical functioning	Emotional functioning	Social functioning	School functioning
1	It is hard for me to walk more than one block	I feel afraid or scared	I have trouble getting along with other kids	It is hard to pay attention in class
2	It is hard for me to run	I feel sad or blue	Other kids do not want to be my friend	I forget things
3	It is hard for me to do sports activity or exercise	I feel angry	Other kids tease me	I have trouble keeping up with my schoolwork
4	It is hard for me to lift something heavy	I have trouble sleeping	I cannot do things that other kids my age can do	I miss school because of not feeling well
5	It is hard for me to take a bath or shower by myself	I worry about what will happen to me	It is hard to keep up when I play with other kids	I miss school to go to the doctor or hospital
6	It is hard for me to do chores around the house			
7	I hurt or ache			
8	I have low energy			

^a^ These were reversed in the Rasch analysis such that 0 = Almost always and 4 = Never

For scoring, items are reverse-scored and linearly transformed onto a numerical 0-100 scale (0 = 100, 1 = 75, 2 = 50, 3 = 25, 4 = 0). Four “multidimensional scales” (Physical Functioning (8 items), Emotional Functioning (5 items), Social Functioning (5 items) and School Functioning (5 items)) can be derived. These can also be combined into three “summary scores” (Total Scale Score (23 items); Physical Health Summary Score (8 items); Psychosocial health Summary Score (15 items)). For all scores higher scores indicate better HRQoL [7, 50]. The Rasch-based analyses in this study were undertaken on the 4 “multidimensional scales” as these form the conceptual basis of all scores derived from PedsQL GCS. The analyses used ordinal responses rather than the transformed numerical 0-100 scale. For ease of interpretation, the ordinal responses were reverse-scored such that higher ordinal scores implied better HRQoL for the given item [37].

### Analyses based on Rasch measurement theory

All Rasch-based analyses were conducted using RUMM2030 software [51]. A random sample of size *N* = 500 was drawn from the eligible CheckPoint sample (*n* = 1,874 children), as this is the recommended sample size to avoid type I error [52–54]. Analyses were repeated for three further random samples of size *N* = 500, drawn from the CheckPoint sample, for cross-validation. Analyses based on Rasch measurement theory use either the rating scale model or the partial credit model. Both models give the same response structure and simply differ in the number of parameters estimated [55]. The partial credit model allows the thresholds for each item to be estimated separately and was used in this analysis. This was deemed appropriate for the diagnostic analysis evaluating the performance of each item’s response scale. As the two models differ only in the number of parameters estimated, we refer to the partial credit model as the Rasch model hereafter [25]. The model was fitted separately for each of the four PedsQL GCS subscales. The criteria described below were assessed [25, 54].

#### Unidimensionality

The Rasch model is a unidimensional measurement model, i.e., it assumes that the individual items of a given PROM form a unidimensional scale when summed together. This assumption was tested by applying a principal component analysis on the residuals to determine if there are other identifiable dimensions in the data after the main ‘Rasch dimension’ has been considered. If there is no interpretable pattern in the residuals, then the unidimensionality assumption can be supported. Two subsets of items were created from the highest and lowest loadings on the first principal component, and a series of independent *t*-tests for each person was used to investigate whether the estimates for those two subsets differed significantly (percentage of individual *t*-tests outside the range +/- 1.96) [56]. If statistically significant differences are found for more than 5% of the sample respondents (specifically, the 95% confidence interval (CI) of the estimated percentage should contain 5%), the assumption of unidimensionality is no longer reasonable.

#### Model goodness of fit

Overall model goodness of fit was tested using the χ^2^ test for item-trait interaction. This divided the respondents into subgroups (class intervals) based on their latent scale levels. Observed and expected responses should be sufficiently similar such that the *P*-value for the χ^2^ test statistic comparing their difference should be > 0.05 in a well-fitting model. In addition, the total person and item fit residuals were examined. These estimates, respectively, are the amount of divergence between the observed and expected responses for each respondent and item, subsequently summed over all respondents (person fit residuals) and items (item fit residuals). The residuals are standardised to approximate the *Z*-score, and the mean residual should be approximately zero with a standard deviation (SD) of approximately one, with large deviations from these indicating poor overall model fit.

#### Model reliability

Reliability was assessed using the person separation index (PSI), which is similar to Cronbach’s alpha. A PSI value >0.7 is considered adequate for group level comparisons, though a higher criterion of 0.85 is usually expected for comparisons at the individual level [25].

#### Targeting

Items in a Rasch model should map out a continuum that is relevant to the individuals being measured. Scale-to-sample targeting was evaluated by comparing the spread of person and item (threshold) locations on the person-item threshold distribution graph. The scale range covered by the items should visibly overlap with that covered by the sample.

#### Item fit

Poorly fitting items have fit residuals lying outside the range of +/-2.5. Moreover, a statistically significant finding for the item-level χ^2^ test for item-trait interaction indicates a poorly fitting item. Bonferroni correction for the number of items in the subscale was applied on the base-case significance threshold of *P*-value < 0.01. Where the item fit was poor, the nature of its fit was examined graphically by inspecting its item characteristic curve.

#### Threshold ordering

The item response levels should sequentially show the highest probability of endorsement, in the order of response level severity. That is, the thresholds for each item should be logically ordered. Disordered thresholds mean that respondents are unable to discriminate between response levels, or they may not have understood the response options in the way that was intended. The category probability curve was plotted for the items with disordered thresholds to assess how the disordering occurred.

#### Differential item functioning

Differential item functioning (DIF) describes the situation where groups of respondents positioned closely together on the latent scale report significantly different item responses. DIF can be uniform or non-uniform depending on whether the difference between groups defined by a ‘person factor’ (across which one would not expect a measurement differential) remains constant or varies across the latent scale, respectively, and can be identified using a series of item-level χ^2^ tests. For the latter, the statistical significance threshold of *P*-value < 0.05 with Bonferroni correction was used. The person factors explored for all models were sex and age (within the target age group).

#### Local dependency

Local or response dependency is where items are linked somehow, such that the response on one item will determine and bias the response on another. Such dependency can be identified through the residual correlation matrix. A pair of items is deemed locally dependent if its positive residual correlation is 0.2 above the average of all item residual correlations within the item set [57].

## Results

### Sample characteristics

Table 2 shows the sample characteristics for the CheckPoint sample and the random sample of size *N* = 500 that was used for Rasch analyses. Table A1 in the Supplementary Information shows the PedsQL GCS item-level responses from the random sample.

**Table 2:** Sample characteristics

	CheckPoint sample [*N* = 1,874]	Random sample used for Rasch analysis [*N* = 500]
Mean age (SD) [Range]	11.5 (0.5) [11–12]	11.5 (0.5) [11–12]
Female N (%)	919 (49.0)	236 (47.2)
SEIFA disadvantage quintile N (%)		
* Most disadvantaged* * 2nd most* * Middle* * 2nd least* * Least disadvantaged*	156 (8.3) 277 (14.8) 351 (18.7) 439 (23.4) 651 (34.7)	42 (8.4) 73 (14.6) 77 (15.4) 126 (25.2) 182 (36.4)
Remoteness area ABS		
* Major cities* * Inner regional* * Outer regional* * Remote* * Very remote* * Missing*	1,318 (70.3) 379 (20.2) 162 (8.6) 9 (0.5) 6 (0.3) 0 (0.0)	353 (70.6) 99 (19.8) 45 (9.0) 3 (0.6) 0 (0.0) 0 (0.0)
Mean BMI (SD)	19.2 (3.4)	19.3 (3.7)
Asthma N (%)	252 (13.5)	67 (13.4)
Diabetes N (%)	7 (0.4)	1 (0.2)
Hearing loss N (%)	22 (1.2)	5 (1.0)
Vision loss N (%)	12 (0.6)	5 (1.0)
Chronic pain N (%)	39 (2.1)	11 (2.2)

Abbreviation: ABS: Australian Bureau of Statistics; BMI: body mass index; SD: standard deviation; SEIFA: socio-economic indexes for areas

### Rasch analysis results

#### Unidimensionality

Table 3 shows the results of the unidimensionality assessment for the Rasch models fitted on each PedsQL GCS subscale. The assumption of unidimensionality appeared reasonable for the physical, emotional, and social functioning subscales but not for school functioning. For the latter, 8.4% of the respondents had statistically significant differences in the positive and negative loading factors after accounting for the main factor, with the 95% confidence interval lying above the 5% (6.4% to 10.4%) threshold.

**Table 3:** Results on Rasch model unidimensionality, goodness of fit, and reliability

Model^a^	Unidimensional? % with significant latent scale difference (95% CI)	Total item fit residual: mean (SD)	Item-trait interaction: χ^2^ test *P*-value	PSI
Physical functioning	Yes: 3.2 (1.1–5.2)	-0.901 (2.032)	*P* < 0.001	0.681
Emotional functioning	Yes: 4.4 (2.4–6.4)	0.221 (1.383)	*P* = 0.152	0.760
Social functioning	Yes: 5.2 (2.9–7.5)	-0.038 (1.309)	*P* = 0.009	0.655
School functioning	No: 8.4 (6.4–10.4)	-0.388 (1.473)	*P* = 0.014	0.714

^a^ Prior to any model adjustment for disordered threshold, local dependency, or differential item functioning

**Abbreviation**: CI: confidence interval; PSI: person separation index; SD: standard deviation

#### Model goodness of fit

Table 3 presents the total item fit residuals and the *P*-value for the χ^2^ test assessing the item-trait interaction for each Rasch model. The *P*-value was above 0.05 (indicating a well-fitting model) only for the emotional functioning subscale. The divergence of the total item fit residual mean and SD from 0 to 1, respectively, was greatest for the physical functioning subscale.

#### Model reliability

The PSI values shown in Table 3 were above 0.7 (indicating adequate model reliability for group level comparisons) for the emotional (0.760) and school (0.714) functioning subscales.

#### Targeting

**Fig. 1:**
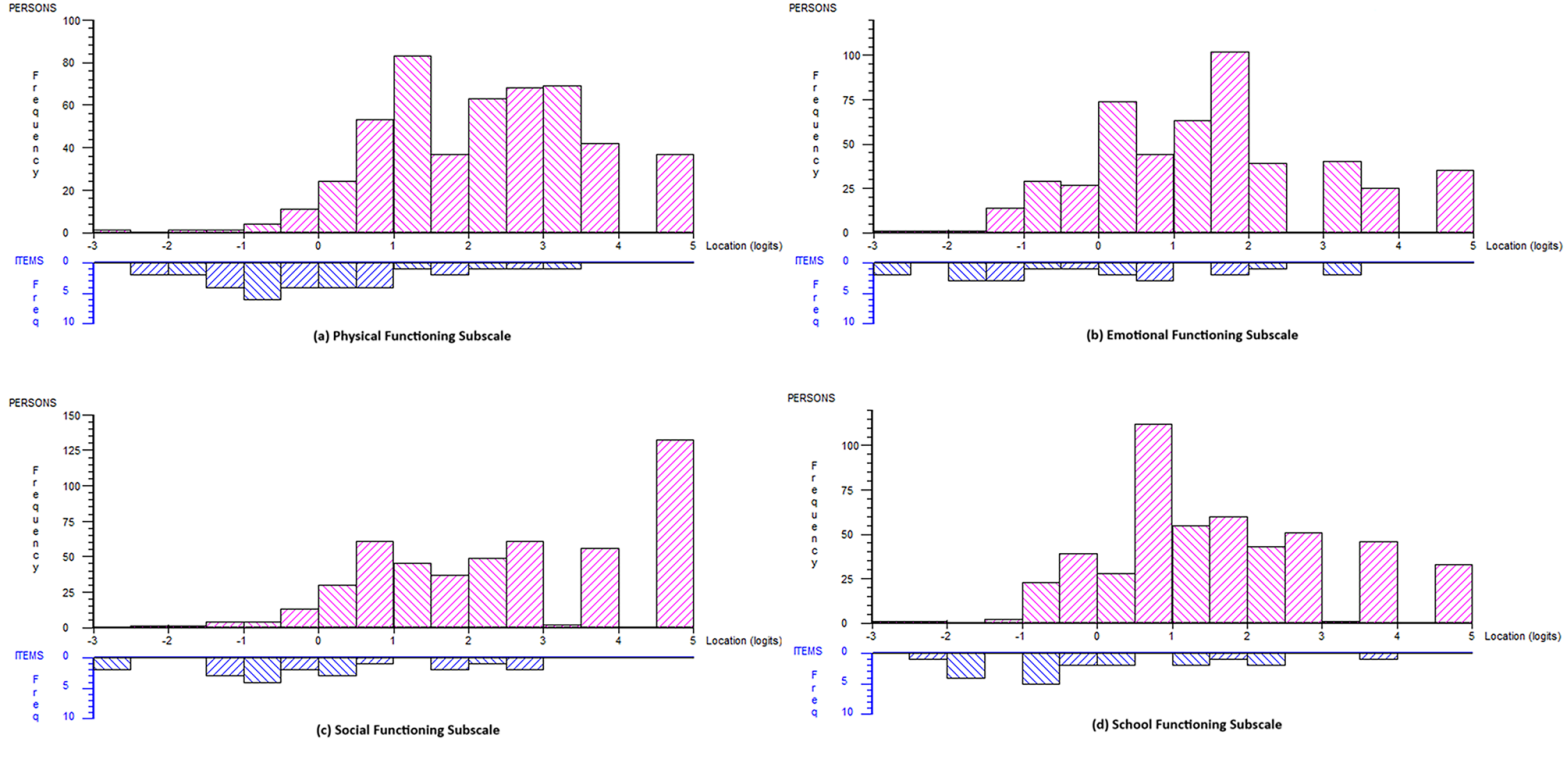
Person-item threshold distribution graphs for (**a**) physical, (**b**) emotional, (**c**) social, and (**d**) school functioning subscales. Note: The y-axis scale differs between subscales according to persons frequency

Figure 1 shows the person-item threshold distribution graphs for each PedsQL GCS subscale, with person (pink bars) and item (blue bars) distributions in the upper and lower halves of the graph, respectively. For all subscales, the person locations were concentrated between 0 and 5 logits on the latent scale (higher position on the scale implied better HRQoL), whereas the item thresholds were generally concentrated between − 2 and 3 on the logit scale. There were, therefore, few items representing the experience of children at the higher end of the scale with better HRQoL. Scores for these children are therefore likely to be less precise. There are also items at the lower end of the scale that may be capturing HRQoL that is too severe to be relevant to this Australian population.

**Table 4:** Results on item-level performance

Item^a^	Item mean location	Threshold range	Item fit residual^b^	Item χ^2^ test significant?^c^	Disordered threshold?	DIF^d^	Local dependency^e^
**Physical functioning subscale**							
1. Walking	-0.304	-0.816 to 0.233	-1.244		Yes		
2. Running	-0.217	-1.871 to 1.337	**-3.444**	Yes			With item 3
3. Sports or exercise	-0.377	-1.471 to 0.939	**-4.363**	Yes			With item 2
4. Lifting something heavy	0.559	-0.637 to 2.754	0.106				
5. Bath or shower	-0.693	-2.053 to 0.597	-0.069		Yes		
6. Doing chores	0.093	-1.333 to 1.746	0.330		Yes	Uniform (sex)	
7. Hurts or aches	0.993	-1.001 to 3.331	1.669				
8. Low energy level	-0.053	-2.062 to 2.421	-0.192			Uniform (age)	
**Emotional functioning subscale**							
1. Afraid or scared	-0.123	-1.940 to 2.431	-0.379		Yes		
2. Sad or blue	-0.207	-2.750 to 3.288	-1.234				With item 3
3. Angry	0.016	-2.606 to 3.148	1.022			Uniform (sex)	With item 2
4. Trouble sleeping	0.356	-1.416 to 1.941	2.217				
5. Worrying	-0.042	-1.510 to 1.830	-0.520				
**Social functioning subscale**							
1. Getting along with other children	-0.146	-2.752 to 2.975	0.179				
2. Other children not friends	-0.146	-2.929 to 2.461	-1.511				
3. Getting teased	0.163	-0.975 to 1.917	0.326		Yes		
4. Cannot do things other children do	0.368	-1.156 to 2.859	1.840				With item 5
5. Keeping up with other children	-0.239	-1.169 to 1.610	-1.025				With item 4
**School functioning subscale**							
1. Paying attention	-0.0002	-1.740 to 2.191	-0.256				With item 3
2. Forgetting things	0.658	-1.679 to 3.697	-1.114			Uniform (age, sex)	
3. Keeping up with schoolwork	-0.105	-2.295 to 1.982	-2.467				With item 1
4. Missing school due to illness	-0.156	-1.645 to 2.022	0.603		Yes		With item 5
5. Missing school to see doctor or hospital	-0.397	-1.560 to 1.070	1.295		Yes		With item 4

^a^ See Table 1 for the full item wordings

^b^ Fit residuals outside the range of -2.5 and 2.5 are highlighted in bold

^c^ Using threshold *P*-value of < 0.01 and Bonferroni correction

^d^ Based on item-specific χ^2^ test with threshold *P*-value of < 0.05 and Bonferroni correction

^e^ A pair of items is deemed locally dependent if its positive residual correlation is 0.2 above the average of all item residual correlations within the item set [57]

Abbreviation: DIF: differential item functioning

#### Item fit

**Fig. 2:**
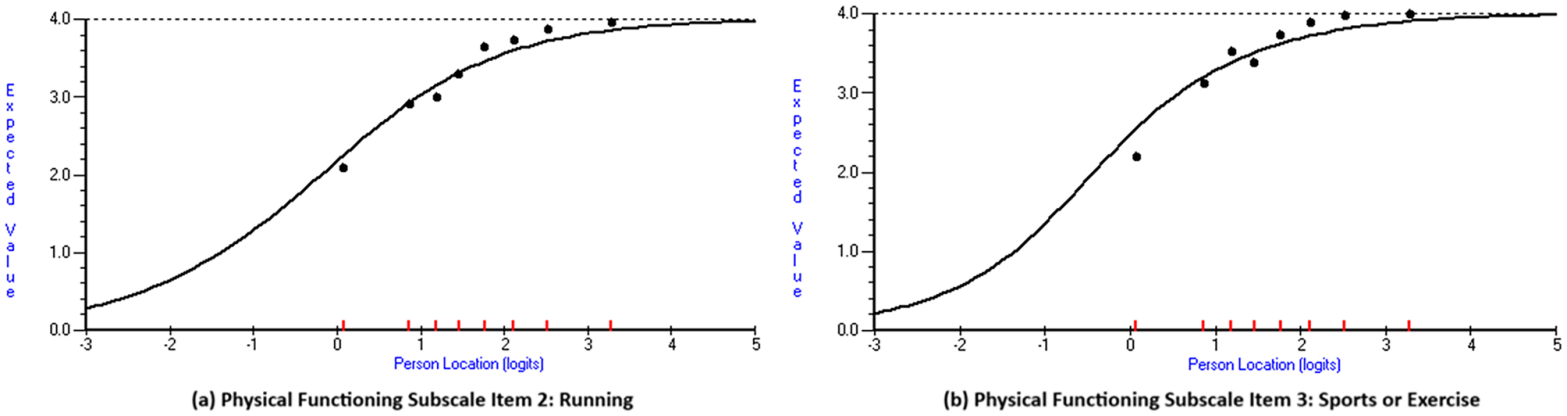
Item characteristic curves for physical functioning subscale items 2 and 3 with poor fit

Table 4 presents the results of the assessments on item-level performance within each Rasch model. Only two of the 23 items had fit residuals lying outside the range of +/-2.5, specifically below -2.5, as well as statistically significant item-level χ^2^ test *P*-values: physical functioning items 2 (running) and 3 (sports or exercise). Figure 2 shows the item characteristic curves for these two items. For both, the observed data (black dots) form a steeper curve than expected by the Rasch model (black solid line). The items were thus over-discriminating, whereby a change in respondents’ position on the latent scale led to greater item score change than the Rasch model expectation.

#### Threshold ordering

**Fig. 3:**
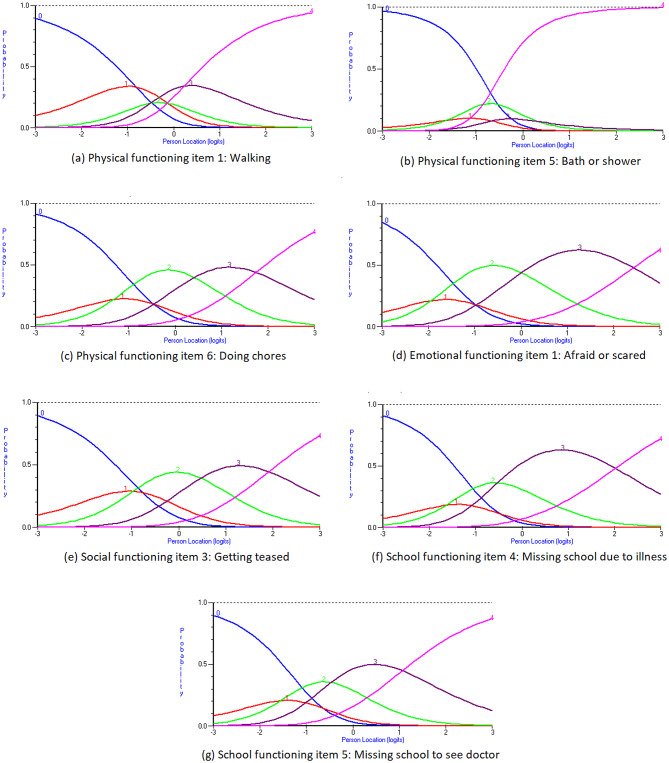
Category probability curves for items with disordered thresholds

Table 4 reports seven items that had disordered thresholds: physical functioning items 1 (walking), 5 (bath or shower) and 6 (doing chores); emotional functioning item 1 (afraid or scared); social functioning item 3 (getting teased); and school functioning items 4 (missing school due to illness) and 5 (missing school to see doctor or hospital). Figure 3 displays the category probability curves for these seven items and shows how the disordering occurred. For six of the seven items, response level 1 (corresponding to having problems ‘Often’) was disordered. For physical functioning item 1 (walking), level 3 (‘Sometimes’) was disordered, while for levels 1–3 were all disordered.

#### Differential item functioning

Table 4 reports four items that had uniform DIF: physical functioning item 6 (doing chores) and emotional functioning item 3 (angry) had DIF by sex; physical functioning item 8 (low energy level) had DIF by age; and school functioning item 2 (forgetting things) had DIF by both age and sex. No item had non-uniform DIF.

#### Local dependency

The identified local dependencies are reported in Table 4. These were between physical functioning items 2 (running) and 3 (sports or exercise); emotional functioning items 2 (sad or blue) and 3 (angry); social functioning items 4 (cannot do things other children do) and 5 (keeping up with other children); and school functioning items 1 (paying attention) and 3 (keeping up with schoolwork), and items 4 (missing school due to illness) and 5 (missing school to see doctor or hospital).

### Cross-validation results

Tables A2 to A5 in the Supplementary Information show the results of cross-validation using three further random samples, presented by each of the four subscales; Figures A1 to A3 show the person-item distribution graphs for the further samples. The results were broadly comparable across the main random sample discussed above and the further samples. Specifically, targeting was very similar for all three random samples across all 4 subscales, the results for the item fit and local dependency criteria were identical across the samples, while some slight variations in threshold ordering and DIF were observed. For example, in the main sample, uniform DIF was observed for item 6 by sex and item 8 by age for the physical functioning subscale, while only uniform DIF for item 6 by sex was observed in the first and second further samples.

## Discussion

This paper is the first to use Rasch-based methods to evaluate the psychometric performance of the child self-report version of the PedsQL GCS in a sample of Australian schoolchildren. It found mixed results. None of the four subscales met al.l of the criteria. The targeting illustrated that all of the subscales had items that described functioning that was too impaired and all four subscales also had too few items representing higher functioning at the higher end, indicating that scores representing higher HRQoL scores were likely to be less precise. Furthermore, although two of the sub-scales (emotional functioning and school functioning) met the PSI (reliability) criterion for group-level comparisons and the remaining two scales (physical functioning and social functioning) approached the criterion, none met the higher criterion required for individual-level comparisons. Overall, the emotional functioning subscale performed best, demonstrating overall fit to the model, sufficient evidence to support unidimensionality, PSI value above 0.7 indicating adequate model reliability, and all items fitting the model. However, this subscale also had one item with disordered thresholds, one item with uniform DIF and one pair of items showing local dependency. Across all subscales, several items did not meet criteria with disordered thresholds (7/23), DIF (4/23), and local dependence (5 pairs of items), though it is often possible to address these issues by post-hoc statistical adjustment [29, 54, 58]. The results were broadly comparable across the cross-validation samples, though slightly different combinations of items had disordered thresholds and DIF; the results on item fit and local dependency were identical across samples.

These findings are consistent with previous studies. Kook and Varni [36] found disordered thresholds for social functioning items in both the self- and proxy-report versions. Other Rasch analyses for the self-reported PedsQL GCS likewise found disordered thresholds, low model reliability, poor targeting, and/or misfitting items [39, 41, 42]; similar issues were found for proxy-report versions [37, 40], though one study noted that targeting improved for children with life-threatening conditions relative to those without [38]. Consistent with our findings on targeting, other authors have also suggested that the focus of the PedsQL GCS on problems or deficits to HRQoL makes it less discriminating for HRQoL differences in healthy children with no conditions that cause significant HRQoL deficits [36], and one suggestion has been to add further items for the higher end of the HRQoL scale [40]. However, ceiling effects may be an inevitable feature for generic HRQoL measures designed to be applied in diverse populations [36]. Previous work has also suggested that the issue of disordered thresholds and response options not working as intended could potentially be resolved by reducing the number of response options from five to three [39–42], though care would be needed to choose appropriate labels for each of the response options.

A key consideration in deciding the psychometric adequacy of a scale is whether it is fit for purpose. To that end, it is important to be clear about the purpose of using the scale. If the purpose is to use individual scores in routine, clinical contexts, then it is important that modern psychometric criteria are met (such as those described by the Rasch paradigm) and that scores are derived based on these models. The published scoring for PedsQL GCS is based on classical psychometric principles and has been validated according to these principles to be appropriate for group-level comparisons [19–24].

There is nevertheless evidence (including this paper) to suggest that the subscales of the PedsQL GCS may not yet meet the more stringent standards that are required for robust measurement at the individual level. Although some of the anomalies identified in this paper such as disordered thresholds, uniform DIF and local dependency could be solved statistically using post-hoc adjustments, the targeting for all four subscales suggests there may be content that is missing particularly towards the higher end of the scale. As we have not undertaken similar analyses of the three summary scores, making these adjustments now would be premature. Further psychometric work is needed to consider whether the pattern is similar for the summary scores, and if so, further qualitative work is needed to consider the nature of the items that might be missing at the higher end of each scale.

It is noteworthy that the issues identified with PedsQL GCS differ between cultural contexts. For example, the current study of Australian children found disordered thresholds in seven items across all four subscales, whereas Kook and Varni’s study [36] of Korean children found disordered thresholds in the social functioning subscale only. Therefore, not only are context- and population-specific psychometric validation warranted to inform any post-hoc modification [29], but also international studies where equating could be considered. These are important topics for research and discussion by the community of PedsQL GCS developers and users. An important consideration would be the need to create cross-walk strategies between legacy and new scoring where post-hoc modification is applied.

As noted, this study is the first to conduct Rasch analysis of the PedsQL GCS in an Australian setting, following established steps in the process [25, 54]. There are, nevertheless, limitations. First, separate Rasch analyses for subgroups of children with and without long-term conditions, such as asthma, and chronic pain was not undertaken. This contrasts with previous studies that compared the Rasch performance between children with and without refractive errors [37] or life-threatening conditions [38]. However, given the aim of the PedsQL GCS of providing a generic measure of HRQoL, subgroup analysis would not necessarily yield further insights to this end [59]. Second, the study was limited by the available data which only included the Child self-report version of the PedsQL GCS. Further research, pending appropriate data, should explore the differences across age versions and between self- and proxy-report versions. Third, we did not perform the Rasch analysis on the total score of PedsQL GCS that combines the subscale-level scores. However, given the multidimensionality of PedsQL GCS, it is unlikely that the unidimensionality assumption required for Rasch analysis would be satisfied. Finally, we did not make post-hoc modifications to the PedsQL GCS according to the Rasch results since our aim was to provide diagnostic evidence on the current performance of the PedsQL GCS, not to develop a modified version.

## Conclusion

Our Rasch-based psychometric evaluation of the PedsQL GCS Child self-report version applied in general population Australian children aged 11–12 years identified important areas where further improvements in the psychometric performance of the PedsQL GCS might be possible, particularly to facilitate use for individual comparisons. However, the Rasch-based psychometric results should be interpreted alongside the conceptual, practical, and classical psychometric strengths of the PedsQL GCS. Further studies using Rasch-based analyses on large international samples are needed.

## Supplementary Information

Below is the link to the electronic supplementary material.

Supplementary Material 1

## Data Availability

Data from the Longitudinal Study of Australian Children (LSAC), including the CheckPoint data used in this study, are available by application to the data custodians: Longitudinal Studies, Data Strategy Branch, Australian Government Department of Social Services.

## Code availability

The STATA DO files are available from the corresponding authors on reasonable request.

## Abbreviations

DIF: Differential item functioning
HRQoL: Health-related quality of life
LSAC: Longitudinal Study of Australian Children
PedsQL GCS: Pediatric Quality of Life Inventory 4.0 Generic Core Scale
PROM: Patient-reported outcome measure
PSI: Person separation index
SD: Standard deviation
