# Supplementary material for: Rasch analysis of the self-reported PedsQL™ 4.0 Generic Core Scales by Australian children
Source: Health Qual Life Outcomes. 2025 Dec 15;23:120. doi: 10.1186/s12955-025-02441-4 (PMC12706910; doi:10.1186/s12955-025-02441-4)
Supplement: Supplementary file 1 — Supplementary Material 1 [file 12955_2025_2441_MOESM1_ESM.docx]

# Rasch analysis of PedsQL™ 4.0 Generic Core Scales self-reported by Australian children

**Supplementary information**

**Authors:**

Dr Joseph Kwon,^1*^ [joseph.kwon@phc.ox.ac.uk](mailto:joseph.kwon@phc.ox.ac.uk); ORCID 0000-0002-2860-7280

Dr Rakhee Raghunandan,^2^ [rakhee.raghunandan@sydney.edu.au](mailto:rakhee.raghunandan@sydney.edu.au); ORCID 0000-0001-7848-6109

Assoc Prof Son Hong Nghiem,^3^ [h.nghiem@uq.edu.au](mailto:h.nghiem@uq.edu.au); ORCID 0000-0002-2451-5290

Prof Kirsten Howard,^2^ [kirsten.howard@sydney.edu.au](mailto:kirsten.howard@sydney.edu.au); ORCID 0000-0002-0918-7540

Prof Emily Lancsar,^4^ [emily.lanscar@anu.edu.au](mailto:emily.lanscar@anu.edu.au); ORCID 0000-0003-2404-6735

Dr Elisabeth Huynh,^4^ [elisabeth.huynh@anu.edu.au](mailto:elisabeth.huynh@anu.edu.au); ORCID 0000-0002-1855-3143

Assoc Prof Martin Howell,^2^ [martin.howell@sydney.edu.au](mailto:martin.howell@sydney.edu.au); ORCID 0000-0001-9740-712X

Prof Stavros Petrou,^1^ [stavros.petrou@phc.ox.ac.uk](mailto:stavros.petrou@phc.ox.ac.uk); ORCID 0000-0003-3121-6050

Assoc Prof Sarah Smith,^5^ [sarah.smith@lshtm.ac.uk](mailto:sarah.smith@lshtm.ac.uk); ORCID 0000-0002-2013-6963

^1^ Nuffield Department of Primary Care Health Sciences, University of Oxford, Oxford, England; ^2^ The Leeder Centre for Health Policy, Economics and Data, School of Public Health, University of Sydney, Sydney, Australia; ^3^ Centre for Health Services Research, University of Queensland, Brisbane, Australia; ^4^ Department of Health Services Research and Policy, Australian National University, Canberra, Australia; ^5^ Department of Health Services Research and Policy, London School of Hygiene and Tropical Medicine, London, England.

* Corresponding author

# PedsQL GCS item-level responses

| **Table A1** PedsQL GCS Children self-report version item-level responses from random sample used for Rasch analysis (N=500). | | | | | | | | | |
| --- | --- | --- | --- | --- | --- | --- | --- | --- | --- |
| **Subscale** | **Item^a^** |  | Never | Almost never | Sometimes | Often | Almost always | Missing, unclear | **Total** |
| Physical | 1. Walking more than one block | N | 396 | 70 | 15 | 7 | 4 | 8 | 500 |
|  |  | % | 79.2 | 14.0 | 3.0 | 1.4 | 0.8 | 1.6 | 100.0 |
|  | 2. Running | N | 311 | 123 | 43 | 11 | 3 | 9 | 500 |
|  |  | % | 62.2 | 24.6 | 8.6 | 2.2 | 0.6 | 1.8 | 100.0 |
|  | 3. Participating in sports or exercise | N | 350 | 104 | 26 | 8 | 3 | 9 | 500 |
|  |  | % | 70.0 | 20.8 | 5.2 | 1.6 | 0.6 | 1.8 | 100.0 |
|  | 4. Lifting something heavy | N | 172 | 217 | 81 | 16 | 6 | 8 | 500 |
|  |  | % | 34.4 | 43.4 | 16.2 | 3.2 | 1.2 | 1.6 | 100.0 |
|  | 5. Taking a bath or shower | N | 471 | 11 | 6 | 1 | 3 | 8 | 500 |
|  |  | % | 94.2 | 2.2 | 1.2 | 0.2 | 0.6 | 1.6 | 100.0 |
|  | 6. Doing chores | N | 270 | 156 | 55 | 7 | 4 | 8 | 500 |
|  |  | % | 54.0 | 31.2 | 11.0 | 1.4 | 0.8 | 1.6 | 100.0 |
|  | 7. Having hurts or aches | N | 103 | 197 | 149 | 35 | 8 | 8 | 500 |
|  |  | % | 20.6 | 39.4 | 29.8 | 7.0 | 1.6 | 1.6 | 100.0 |
|  | 8. Low energy level | N | 206 | 201 | 72 | 12 | 1 | 8 | 500 |
|  |  | % | 41.2 | 40.2 | 14.4 | 2.4 | 0.2 | 1.6 | 100.0 |
| Emotional | 1. Feeling afraid or scared | N | 147 | 232 | 95 | 12 | 7 | 7 | 500 |
|  |  | % | 29.4 | 46.4 | 19.0 | 2.4 | 1.4 | 1.4 | 100.0 |
|  | 2. Feeling sad or blue | N | 98 | 246 | 128 | 19 | 3 | 6 | 500 |
|  |  | % | 19.6 | 49.2 | 25.6 | 3.8 | 0.6 | 1.2 | 100.0 |
|  | 3. Feeling angry | N | 94 | 211 | 156 | 28 | 4 | 7 | 500 |
|  |  | % | 18.8 | 42.2 | 31.2 | 5.6 | 0.8 | 1.4 | 100.0 |
|  | 4. Trouble sleeping | N | 154 | 147 | 117 | 60 | 16 | 6 | 500 |
|  |  | % | 30.8 | 29.4 | 23.4 | 12.0 | 3.2 | 1.2 | 100.0 |
|  | 5. Worrying about what will happen | N | 177 | 161 | 118 | 28 | 9 | 7 | 500 |
|  |  | % | 35.4 | 32.2 | 23.6 | 5.6 | 1.8 | 1.4 | 100.0 |
| Social | 1. Getting along with others | N | 230 | 200 | 51 | 14 | 1 | 4 | 500 |
|  |  | % | 46.0 | 40.0 | 10.2 | 2.8 | 0.2 | 0.8 | 100.0 |
|  | 2. Other kids not being friends | N | 256 | 159 | 58 | 18 | 2 | 7 | 500 |
|  |  | % | 51.2 | 31.8 | 11.6 | 3.6 | 0.4 | 1.4 | 100.0 |
|  | 3. Getting teased by other children | N | 290 | 132 | 55 | 11 | 6 | 6 | 500 |
|  |  | % | 58.0 | 26.4 | 11.0 | 2.2 | 1.2 | 1.2 | 100.0 |
|  | 4. Not able to do things that other children can do | N | 226 | 174 | 77 | 13 | 4 | 6 | 500 |
|  |  | % | 45.2 | 34.8 | 15.4 | 2.6 | 0.8 | 1.2 | 100.0 |
|  | 5. Keeping up when playing with children | N | 318 | 131 | 34 | 7 | 3 | 7 | 500 |
|  |  | % | 63.6 | 26.2 | 6.8 | 1.4 | 0.6 | 1.4 | 100.0 |
| School | 1. Paying attention in class | N | 161 | 202 | 96 | 29 | 7 | 5 | 500 |
|  |  | % | 32.2 | 40.4 | 19.2 | 5.8 | 1.4 | 1.0 | 100.0 |
|  | 2. Forgetting things | N | 71 | 202 | 163 | 48 | 9 | 7 | 500 |
|  |  | % | 14.2 | 40.4 | 32.6 | 9.6 | 1.8 | 1.4 | 100.0 |
|  | 3. Keeping up with school activities | N | 175 | 170 | 105 | 37 | 4 | 9 | 500 |
|  |  | % | 35.0 | 34.0 | 21.0 | 7.4 | 0.8 | 1.8 | 100.0 |
|  | 4. Missing school, not feeling well | N | 190 | 231 | 59 | 8 | 5 | 7 | 500 |
|  |  | % | 38.0 | 46.2 | 11.8 | 1.6 | 1.0 | 1.4 | 100.0 |
|  | 5. Missing school, to go to doctor | N | 263 | 169 | 49 | 7 | 4 | 8 | 500 |
|  |  | % | 52.6 | 33.8 | 9.8 | 1.4 | 0.8 | 1.6 | 100.0 |
| ^a^ See Table 1 for the full item wording.  **Abbreviation:** PedsQL GCS: Pediatric Quality of Life Inventory 4.0 Generic Core Scale | | | | | | | | | |

# Cross-validation on additional randomly generated samples

| **Table A2** Cross-validation for physical functioning subscale on additional random samples | | | | |
| --- | --- | --- | --- | --- |
| **Criteria** | **Main sample** | **Sensitivity analyses** | | |
|  |  | **Sample 1** | **Sample 2** | **Sample 3** |
| Uni-dimensionality | 3.2% (95% CI: 1.1-5.2) with significant latent scale difference | 4.8% (95% CI: 2.8-6.7) | 2.7% (95% CI: 0.6-4.7) | 4.4% (95% CI: 2.4-6.4) |
| Model goodness of fit | Item-trait interaction χ^2^ test *P*<0.001 | χ^2^ test *P*<0.001 | χ^2^ test *P*<0.001 | χ^2^ test *P*<0.001 |
| Model reliability | PSI = 0.681 | PSI = 0.687 | PSI = 0.732 | PSI = 0.685 |
| Targeting | See Figure 1 | See Figure A1 | See Figure A2 | See Figure A3 |
| Item fit | Items 2 and 3 with residual outside range +/-2.5 and significant χ^2^ test *P*-value | *Same as main sample* | *Same as main sample* | *Same as main sample* |
| Threshold ordering | Disordered threshold for items 1, 5 and 6 | Disordered threshold for items 1 and 5 | Disordered threshold for items 1, 5, 6 and 8 | Disordered threshold for items 1 and 5 |
| Differential item functioning | Uniform DIF for item 6 by sex and item 8 by age | Uniform DIF for item 6 by sex | Uniform DIF for item 6 by sex | *Same as main sample* |
| Local dependency | Dependency for items 2 and 3 | *Same as main sample* | *Same as main sample* | *Same as main sample* |
| **Abbreviation:** CI: confidence interval; DIF: differential item functioning; PSI: person separation index | | | | |

| **Table A3** Cross-validation for emotional functioning subscale on additional random samples | | | | |
| --- | --- | --- | --- | --- |
| **Criteria** | **Main sample** | **Sensitivity analyses** | | |
|  |  | **Sample 1** | **Sample 2** | **Sample 3** |
| Uni-dimensionality | 4.4% (95% CI: 2.4-6.4) with significant latent scale difference | 6.5% (95% CI: 4.6-8.5) | 6.1% (95% CI: 4.1-8.0) | 6.8% (95% CI: 4.8-8.7) |
| Model goodness of fit | Item-trait interaction χ^2^ test *P*=0.152 | χ^2^ test *P*=0.061 | χ^2^ test *P*=0.020 | χ^2^ test *P*=0.041 |
| Model reliability | PSI = 0.760 | PSI = 0.764 | PSI = 0.746 | PSI = 0.744 |
| Targeting | See Figure 1 | See Figure A1 | See Figure A2 | See Figure A3 |
| Item fit | No item with residual outside range +/-2.5 and significant χ^2^ test *P*-value | *Same as main sample* | *Same as main sample* | *Same as main sample* |
| Threshold ordering | Disordered threshold for item 1 | Disordered thresholds for items 1 and 5 | No disordered threshold | *Same as main sample* |
| Differential item functioning | Uniform DIF for item 3 by sex | *Same as main sample* | *Same as main sample* | *Same as main sample* |
| Local dependency | Dependency for items 2 and 3 | *Same as main sample* | *Same as main sample* | *Same as main sample* |
| **Abbreviation:** CI: confidence interval; DIF: differential item functioning; PSI: person separation index | | | | |

| **Table A4** Cross-validation for social functioning subscale on additional random samples | | | | |
| --- | --- | --- | --- | --- |
| **Criteria** | **Main sample** | **Sensitivity analyses** | | |
|  |  | **Sample 1** | **Sample 2** | **Sample 3** |
| Uni-dimensionality | 5.2% (95% CI: 2.9-7.5) with significant latent scale difference | 3.9% (95% CI: 1.7-6.1) | 4.7% (95% CI: 2.5-6.9) | 6.0% (95% CI: 3.7-8.2) |
| Model goodness of fit | Item-trait interaction χ^2^ test *P*=0.009 | χ^2^ test *P*=0.005 | χ^2^ test *P*=0.011 | χ^2^ test *P*=0.063 |
| Model reliability | PSI = 0.655 | PSI = 0.692 | PSI = 0.665 | PSI = 0.670 |
| Targeting | See Figure 1 | See Figure A1 | See Figure A2 | See Figure A3 |
| Item fit | No item with residual outside range +/-2.5 and significant χ^2^ test *P*-value | *Same as main sample* | *Same as main sample* | *Same as main sample* |
| Threshold ordering | Disordered threshold for item 3 | *Same as main sample* | Disordered threshold for item 1 | *Same as main sample* |
| Differential item functioning | No uniform or non-uniform DIF | *Same as main sample* | *Same as main sample* | *Same as main sample* |
| Local dependency | Dependency for items 4 and 5 | *Same as main sample* | *Same as main sample* | *Same as main sample* |
| **Abbreviation:** CI: confidence interval; DIF: differential item functioning; PSI: person separation index | | | | |

| **Table A5** Cross-validation for school functioning subscale on additional random samples | | | | |
| --- | --- | --- | --- | --- |
| **Criteria** | **Main sample** | **Sensitivity analyses** | | |
|  |  | **Sample 1** | **Sample 2** | **Sample 3** |
| Uni-dimensionality | 8.4% (95% CI: 6.4-10.4) with significant latent scale difference | 9.5% (95% CI: 7.5-11.5) | 9.5% (95% CI: 7.6-11.5) | 9.4% (95% CI: 7.4-11.3) |
| Model goodness of fit | Item-trait interaction χ^2^ test *P*=0.014 | χ^2^ test *P*=0.005 | χ^2^ test *P*<0.001 | χ^2^ test *P*<0.001 |
| Model reliability | PSI = 0.714 | PSI = 0.680 | PSI = 0.678 | PSI = 0.691 |
| Targeting | See Figure 1 | See Figure A1 | See Figure A2 | See Figure A3 |
| Item fit | No item with residual outside range +/-2.5 and significant χ^2^ test *P*-value | *Same as main sample* | *Same as main sample* | *Same as main sample* |
| Threshold ordering | Disordered threshold for items 4 and 5 | *Same as main sample* | *Same as main sample* | *Same as main sample* |
| Differential item functioning | Uniform DIF for item 2 by sex and age | No uniform or non-uniform DIF | Uniform DIF for item 2 by sex | No uniform or non-uniform DIF |
| Local dependency | Dependency for items 1 and 3 and items 4 and 5 | *Same as main sample* | *Same as main sample* | *Same as main sample* |
| **Abbreviation:** CI: confidence interval; DIF: differential item functioning; PSI: person separation index | | | | |


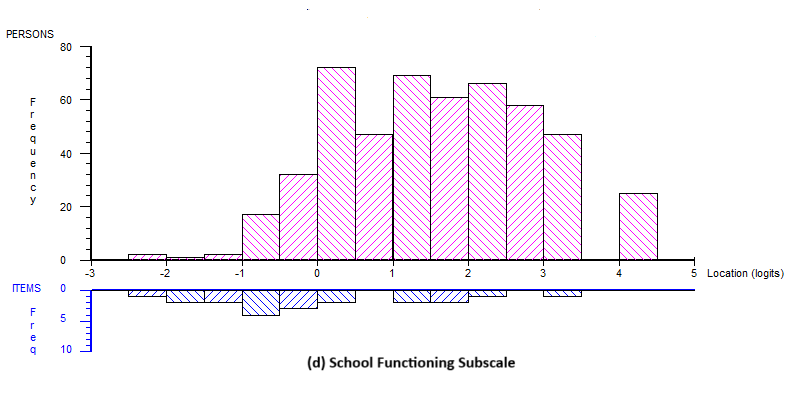

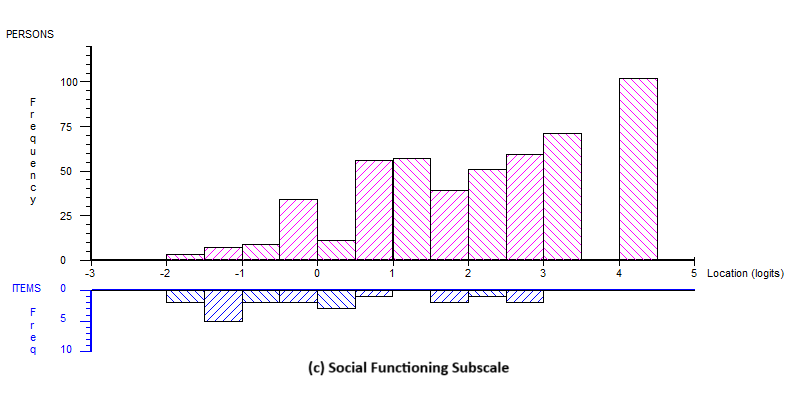

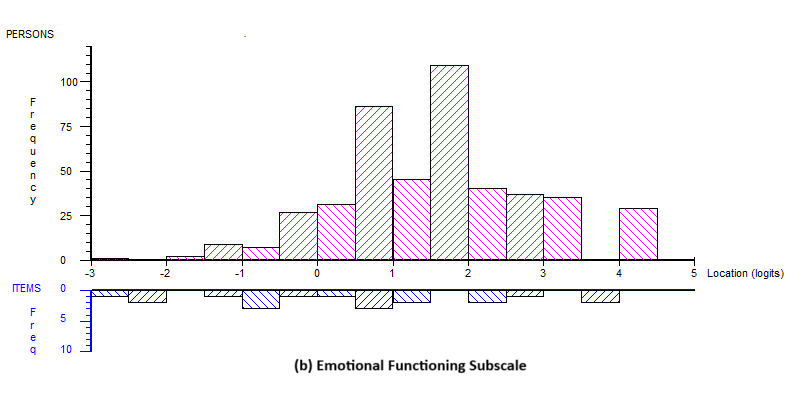

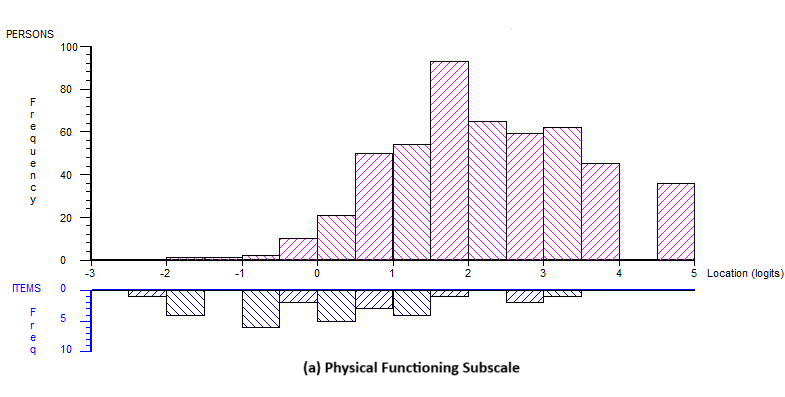
**Figure A1** Person-item threshold distribution graphs by subscale in additional random sample 1. **Note:** The y-axis scale differs between subscales according to persons frequency.


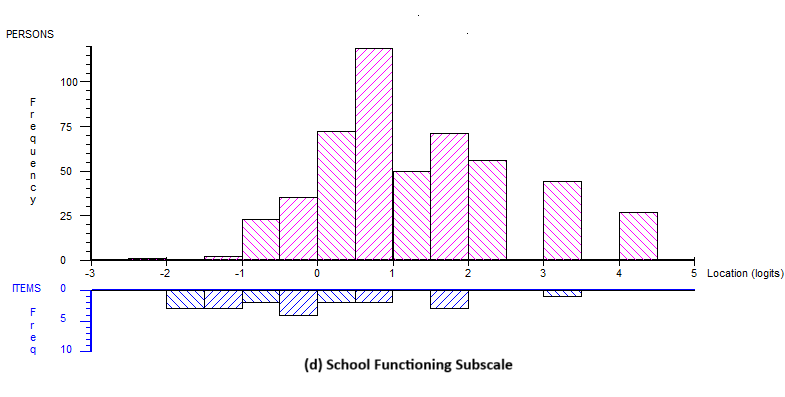

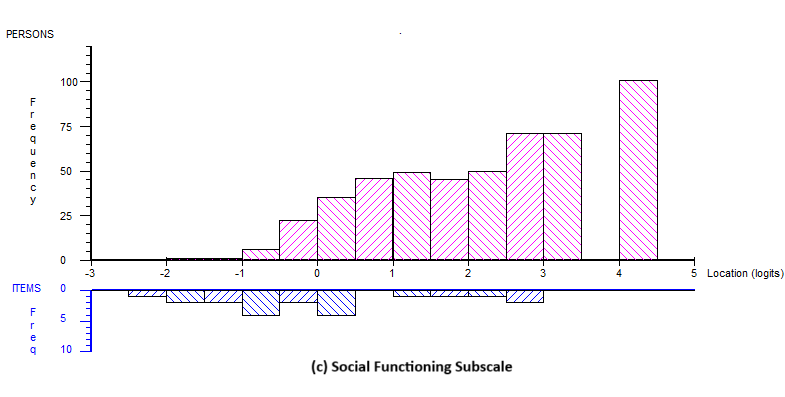

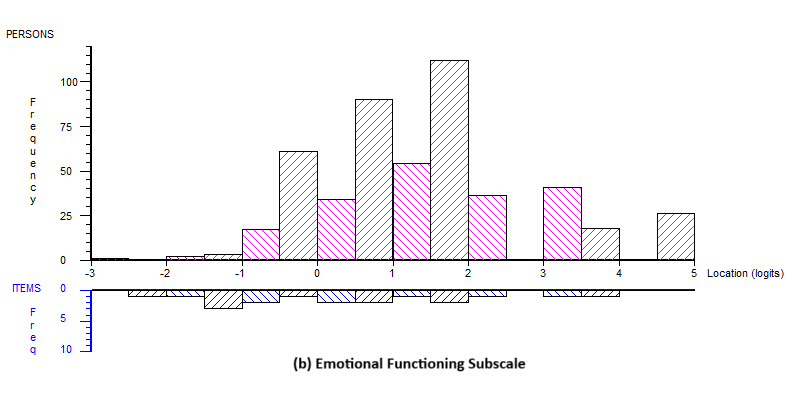

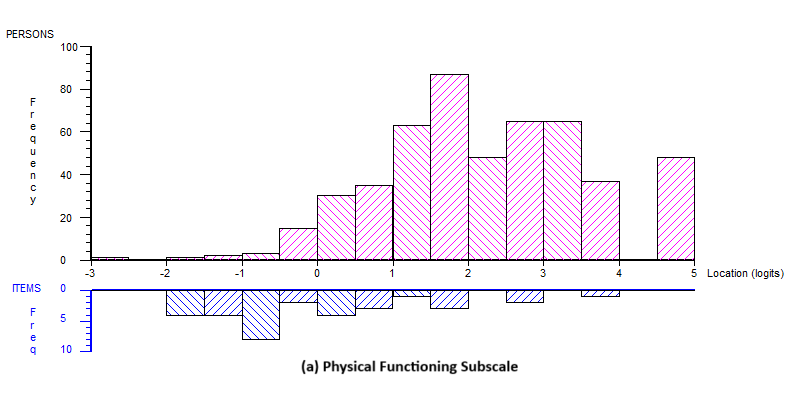


**Figure A2** Person-item threshold distribution graphs by subscale in additional random sample 2. **Note:** The y-axis scale differs between subscales according to persons frequency.


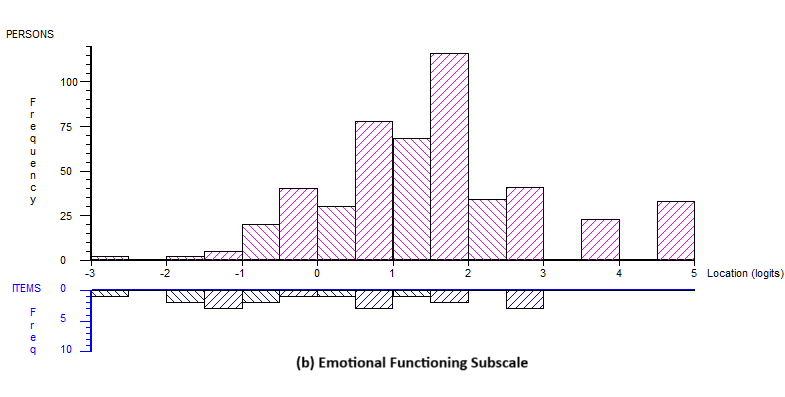

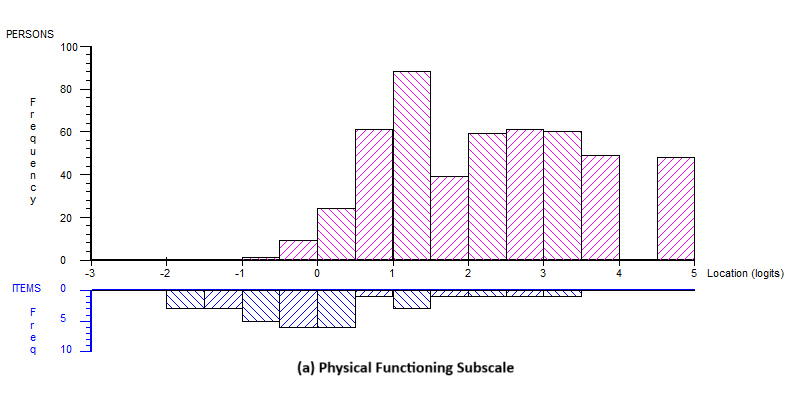


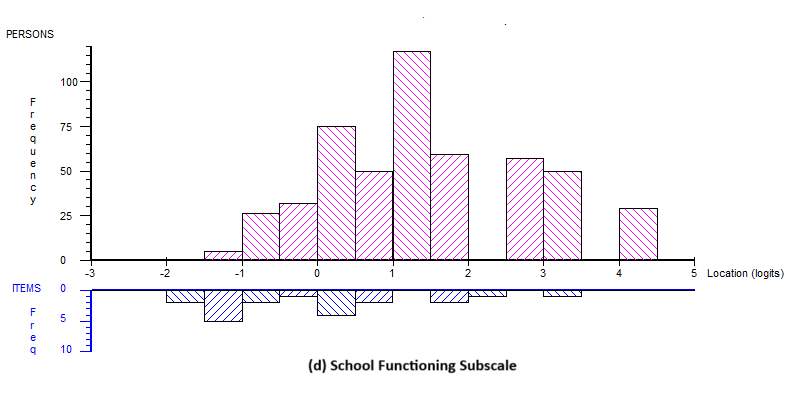

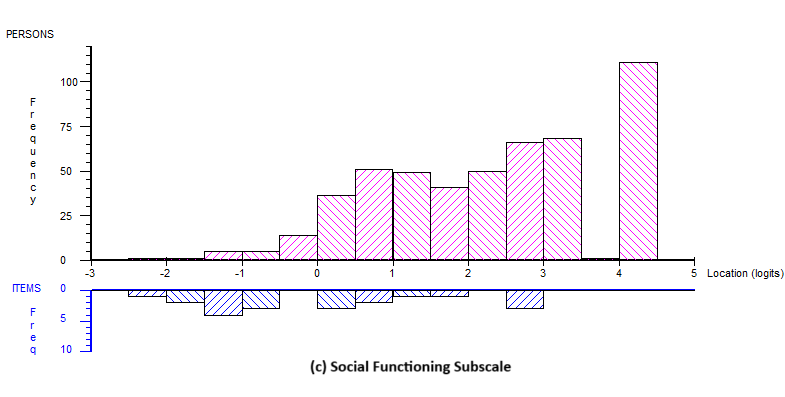


**Figure A3** Person-item threshold distribution graphs by subscale in additional random sample 3. **Note:** The y-axis scale differs between subscales according to persons frequency.
